# Ruminant Placental Adaptation in Early Maternal Undernutrition: An Overview

**DOI:** 10.3389/fvets.2021.755034

**Published:** 2021-10-20

**Authors:** Paola Toschi, Mario Baratta

**Affiliations:** ^1^Department of Veterinary Sciences, University of Turin, Grugliasco, Italy; ^2^Department of Chemistry, Life Sciences and Environmental Sustainability, Viale delle Scienze, University of Parma, Parma, Italy

**Keywords:** placenta, undernutrition, early pregnancy, autophagy, ruminants

## Abstract

Correct placental development during early gestation is considered the main determinant of fetal growth in late pregnancy. A reduction in maternal nourishment occurring across the early developmental window has been linked to a wide range of pregnancy disorders affecting placental transport capacity and consequently the fetal nutrient supply line, with long-term implications for offspring health and productivity. In livestock, ruminant species specifically experience maternal undernutrition in extensive systems due to seasonal changes in food availability, with significant economic losses for the farmer in some situations. In this review, we aim to discuss the effects of reduced maternal nutrition during early pregnancy on placental development with a specific focus on ruminant placenta physiology. Different types of placental adaptation strategies were examined, also considering the potential effects on the epigenetic landscape, which is known to undergo extensive reprogramming during early mammalian development. We also discussed the involvement of autophagy as a cellular degradation mechanism that may play a key role in the placental response to nutrient deficiency mediated by mammalian target of rapamycin, named the mTOR intracellular pathway.

## Introduction

In mammals, the establishment of correct placentation is one of the key steps to ensuring a successful pregnancy outcome. As the major determinant of fetal growth, the placenta is the first organ that forms during embryonic development. It constitutes the foeto-maternal interface, whose primary function is to regulate the exchange of respiratory gases, nutrients, and waste products between the mother and fetus (1). Although the placenta functional role has long been underestimated, placental development was recently examined in a number of studies revealing the existence of a finely regulated control system responsible for the balanced distribution of nutrients to fulfill the increasing demands for the growing fetus without jeopardizing the mother's health (2).

Since placental growth precedes fetal growth, events that occur during early pregnancy, such as trophoblast cell differentiation and placental vascularization, are thought to be particularly critical for correct placentation (1). Any perturbations in these developmental processes affect placental efficiency and in turn compromise the fetal nutrient supply line, leading to pregnancy complications such as intrauterine growth restriction (IUGR) (3). Moreover, a growing body of data obtained from clinical and experimental studies correlates the onset of adult diseases with the occurrence of *in utero* adverse conditions (4). These stress-induced disturbances influence placental development, leading to changes in fetal uptake of nutrients as well as in the secretion of hormones and other signaling molecules into foeto-maternal circulation (4). Furthermore, as an important process of epigenetic remodeling occurs during the early development of mammals (5), an increasing number of articles have highlighted the presence of epigenetic modifications in specific genes as well as in the global genome for offsprings raised under uterine suboptimal conditions (6).

Overall, the nutritional status of the mother is the most important external factor influencing the intrauterine environment (7), with significant implications for fetal growth (8, 9), and long-term health of the offspring (10–12), in a process known as fetal programming. Experiencing suboptimal maternal nutrition can lead the growing fetus to different outcomes depending on the timing and severity of the nutritional insults (13). To date, the effects of reduced maternal nutrition on fetal growth and metabolism as well as susceptibility to the development of cardiovascular or metabolic diseases in adulthood have been discussed in livestock (14) as well as in humans (15). In particular, ruminant species seem to be affected by poor maternal nutrition, which frequently occurs due to seasonal changes in forage quantity and quality. In most cases, if not adequately supplemented, grazing-based diets often fail to provide adequate nutrients and may have a negative impact on the growth and performance of both mothers and offspring, with important economic losses for farmers (16, 17).

This review aims to focus on early pregnancy interactions between mothers and offspring in ruminants to discuss the consequences of reduced maternal nutrition on the development of the placenta and the adaptation strategies that follow. In fact, although the ability of the ruminant placenta to adapt its development to meet fetal growth demands has been previously reported in pregnancies complicated by nutrient deficiencies (18), knowledge about the mechanisms allowing such compensatory action is still fragmentary.

Moreover, we analyzed recent information on the impact of autophagy, a cell survival mechanism that is particularly active under starvation conditions (19), and strengthened the hypothesis that it serves as a rescue mechanism to counterbalance low nutrient availability through the self-digestion of trophoblast cytosolic components and the recycling of obtained nutrients for the benefit of the growing fetus (20). Some evidence in the literature (summarized in Table 1) seems to suggest that autophagy could represent a powerful tool available for the placenta to bypass transitory nutritional stress, even if excessive autophagy beyond certain limits leads to cell death, as reported in many placental pathologies (21, 22).

**Table 1:** References on the role of autophagy on trophoblastic cell function and placental formation.

**Cells /tissues**	**Treatment**	**Autophagy**	**TC effect**	**Model**	**References**
−HTR8/SVneo HUVECs	Oxidative stress	Increased	−↓ TC invasion −↓ endothelial cell tube formation	PE	(21)
−EVT HUVECs	Hypoxia and sENG	Inhibition	−↓ EVT invasion −↓ of vascular remodeling	PE	(117)
−HTR8 JEG3	LncRNA-H19 overexpression	Induction	−↓ TC viability −↑ invasion	PE	(129)
−HTR8 JEG	Silencing LncRNA-H19	Induction	↓ TC proliferation and invasion	FGR	(128)
−HTR8/SVneo JEG3	ATG5 or beclin-1 shRNA	Inhibition	↑ In the invasiveness of TC	PE	(122)
HTR8/SVneo	Rapamycin/Bafilomicin and IGF2 supplementation	Modulation	Modulation of TC invasion	RSA	(121)
Cytotrophoblasts from IUGR placenta	Hypoxia and p53 activation	Modulation	Modulation of TC turnover	IUGR/PE	(118)
HTR8/SVneo	Folate-deficient condition and 3MA	Modulation	Modulation of TC invasion ability and apoptosis	Folate deficiency	(126)
Porcine trophoblast cells (pTr)	ROS inducing condition	Induction	Modulation of TC attachment and differentiation	–	(124)
HTR8/SVneo	LRP6 overexpression under H/R	Induction	↑ TC growth, invasion and migration	PE	(120)
−HTR8/SVneo −HTR8-ATG4BC74A (autophagy-deficient)	HIF1a overexpression	Modulation	Modulate TC homeostasis and invasiveness	Hypoxia, PE	(125)
Mouse placenta (d19)	Gestational food restriction	Decreased	−↓ Vascularity −Damaged TC layers	IUGR	(137)
Mouse placenta (d10-14)	Folate deficient diet	Impaired	−Impaired placental morphology −Hemorrhages −Disturbed TC layer patterning	Folate related diseases	(126)
Mouse placenta (d14.5)	3MA	Inhibition	Increased absorption rate	RSA	(128)
Sheep placenta (d20)	*in vitro* embryo production	Increased	−Impaired placental structure −Disturbed TC layer organization	ART induced defects	(132)
Rat placenta (d16)	Hyperoside	Activated	−Suppressed the inflammation and NF-κB activation	Immune mediated RPL	(136)

*Cells: HTR8/SVneo, human first trimester extravillous trophoblasts; LncRNA, long non coding RNA; shRNA, short hairpin RNA; IGF2, insulin growth factors; HUVECs, human umbilical vein endothelial cells; ROS, reactive oxygen species; JEG3, choriocarcinoma cells; HTR8, human extravillous trophoblast cells; EVT, human extravillous trophoblast cells. Treatments: sENG, soluble endoglin; 3MA, 3Methyladenine; HIF-1α, hypoxia inducible factors; LRP6, low-density lipoprotein receptor-related protein 6; H/R, hypoxia/reoxygenation. Model: PE, preeclampsia; IUGR, intrauterine growth restriction; FGR, fetal growth restriction; RSA, spontaneous recurrent abortion; ART, assisted reproductive technologies; RPL, immune mediated recurrent pregnancy loss*.

## Nutrients Transport Across Ruminant Placenta

### Early Placenta Development

The structure of the placenta is specifically designed to support the survival of the conceptus in the intrauterine environment and represents the juxtaposition of two components with different origins: the uterine endometrium constitutes the maternal portion, while the fetal portion is mainly composed of trophoblast cells, which are the first lineage specified during embryogenesis (1). Soon after fertilization, under a complex mechanism of epigenetic and transcriptional regulation, the mammalian embryo reaches the blastocyst stage and differentiates into two distinct cell lineages: the inner cluster of cells, which later give rise to the proper fetus, and the outermost line of cells surrounding the blastocyst, called the trophoectoderm, which is programmed to give rise exclusively to the population of trophoblast cells, the main cell type of the placental structures (6). At the time of implantation, trophoblast cells differentiate into a variety of trophoblast cell subtypes with different functions, which cooperate to form the mature placenta. Despite the high variability in placental structures and trophoblast cell subtypes between different mammalian species, trophoblast cells follow identical developmental steps during early pregnancy, including proliferation, differentiation, migration and, in some cases, invasion (1).

In ruminant placenta (defined as synepitheliochorial or cotyledonary), the foeto-maternal exchange of nutrients occurs primarily throughout specialized areas of attachment called placentomes, formed by the fusion of fetal cotyledons with endometrial caruncles, randomly distributed to the whole placental tissue (23). In ewes, the growth of the cotyledonary mass is exponential during the first 80 days of pregnancy, while in cows, although there is no change in the number of bovine placentomes, the mean size progressively increases throughout gestation, leading to a greater complexity of the placentome vasculature (24, 25). Ruminant trophoblast cells differentiate into two cell types characterized by different morphologies and functionalities: mononucleated and binucleated cells. The first type is the main structural component of the placenta, which is directly involved in the nutrient exchange process due to its specific morphological features, such as apical microvilli or intracellular junctions. Binucleated cells constitute ~20% of the total population, and their migration and fusion with maternal endometrial cells generate foeto-maternal syncytia and consequently promote implantation and placentome formation. Along with their structural role, binucleated cell functions include hormone production, such as placental lactogen and steroid hormones (23, 26).

During early placental development, along with trophoblast cell differentiation, the establishment of functional vascular architecture determines the placental ability to support the exponential increase in fetal growth later in pregnancy. In fact, a well-developed placental vasculature is necessary to increase the utero-placental blood flow and thus the supply of nutrients and other circulating molecules from maternal to fetal blood (13, 25). At the beginning of placental development, vascularization leads to *de novo* endothelial cell differentiation, followed by self-organization to form capillary-like tubes. Then, starting from this primary vascular network, new capillaries begin to form by angiogenesis (27). Although knowledge of placental vascular development is still fragmentary, the key factors involved have nevertheless been identified during early pregnancy in ruminants and comprise vascular endothelial growth factor (VEGF), angiopoietins (ANG1/ANG2), fibroblast growth factors (FGF), and their respective receptors (28–30). In summary, most of the events responsible for proper placental development occur during early pregnancy, making this development time window particularly important for a well-balanced distribution of nutrients between mother and fetus later in gestation.

### Main Mechanisms of Nutrients Transport

Throughout pregnancy, the developing fetus is dependent on the transplacental supply of nutrients from maternal to fetal circulation. The major substrates required for foeto-placental growth include glucose, amino acids, and fatty acids, and their transport occurs through two main mechanisms of facilitative diffusion that can involve either passive transport down a concentration gradient or active transport processes against a concentration gradient (31). Moreover, as a metabolic organ, the placenta is highly responsive to a wide range of nutritional signals of adversity. Generally, for substances crossing the placenta by passive diffusion, there will be no or reduced transport if the maternal gradient is not maintained, while active transport will also be reduced, as it receives less substrate for energy production (32). More details on the most important transplacental transport mechanisms found under nutrient-deficient conditions in livestock were previously discussed (31–33).

Glucose is the main energy substrate to sustain foeto-placenta development. Its transport occurs down a concentration gradient and is mediated by glucose transporters (GLUTs) (31). In pregnant sheep, placental glucose consumption represents ~75% of the glucose available from the uterine circulation, and this proportion increases with decreasing glucose concentration in the maternal artery (34). A large portion of glucose is used by the placenta to obtain lactate, which is considered a key fuel for fetal growth (35), and its production varies in relation to the rate of placental glucose consumption (36). Moreover, insulin growth factor 1 (IGF1) is known to play an important role as a positive regulator of glucose uptake by stimulating the expression of the GLUT1 receptor, which is the predominant glucose transporter in the placenta (37, 38).

In sheep, reduced maternal nutrition [60% of energy requirements (ER)] from early to mid-gestation (28–80 days) reduces placental mass but not the number of GLUT-1 receptors (39). Similar results have been obtained following short-term (83–90 days) acute maternal restriction during mid-gestation, suggesting that the placental glucose transport system is less affected by nutrient reduction (40).

Normal placental function is also strictly dependent on amino acid availability. Amino acids are both locally synthesized and derived from the maternal circulation using energy-dependent active transport (31). This mechanism is operated by multiple amino acid transporters, whose expression and activity are regulated by several factors, such as amino acid and glucose concentrations and insulin, leptin, and IGF1 levels (41–44). In ruminant species, the developing placenta displays a specific pattern of amino acid production and foeto-maternal distribution according to breed and nutrition (45). In particular, maternal undernutrition (−50%) has often been associated with changes in fetal and maternal amino acid profiles caused by reduced placental transport or metabolism (46, 47). Additionally, the developing fetus and its corresponding placenta require lipids and fatty acids to sustain membrane formation and fetal fat stores. Fatty acids are taken up by the placenta and transported to the fetus from two main sources in the maternal circulation: free fatty acids (FFAs) non-esterified and esterified fatty acids in triglycerides (TGs) carried by lipoproteins (33). FFAs are transported along the concentration gradient by various FFA transporter proteins, whose expression and activity are influenced by insulin, IGF1, and leptin (40, 48). Lipoprotein-bound TGs in the maternal circulation are hydrolyzed by placental lipoprotein lipase to release long-chain fatty acids and allow fatty acid transporter/binding proteins (FATPs/FABPs) to transport them across the placental membranes (49). In sheep, in response to maternal undernutrition, there are changes in the TGs and FFAs profiles of fetal and maternal plasma, which may be related, in part, to modification of placental fatty acids transport or metabolism (40, 50).

Finally, in the case of reduced nutrient availability, the placenta plays a central role in regulating the allocation of resources to the developing fetus, operating at different levels for each transport mechanism.

### Factors Affecting Placental Transport Capacity

Nutrient transport across the placenta depends first on morphological characteristics such as placental shape and size, utero-placental vascularization, and density of transporter proteins. In addition to structural features, the transplacental exchange of nutrients can also be modulated by various signals, including hormones, growth factors, and cytokines (18).

**Figure 1:**
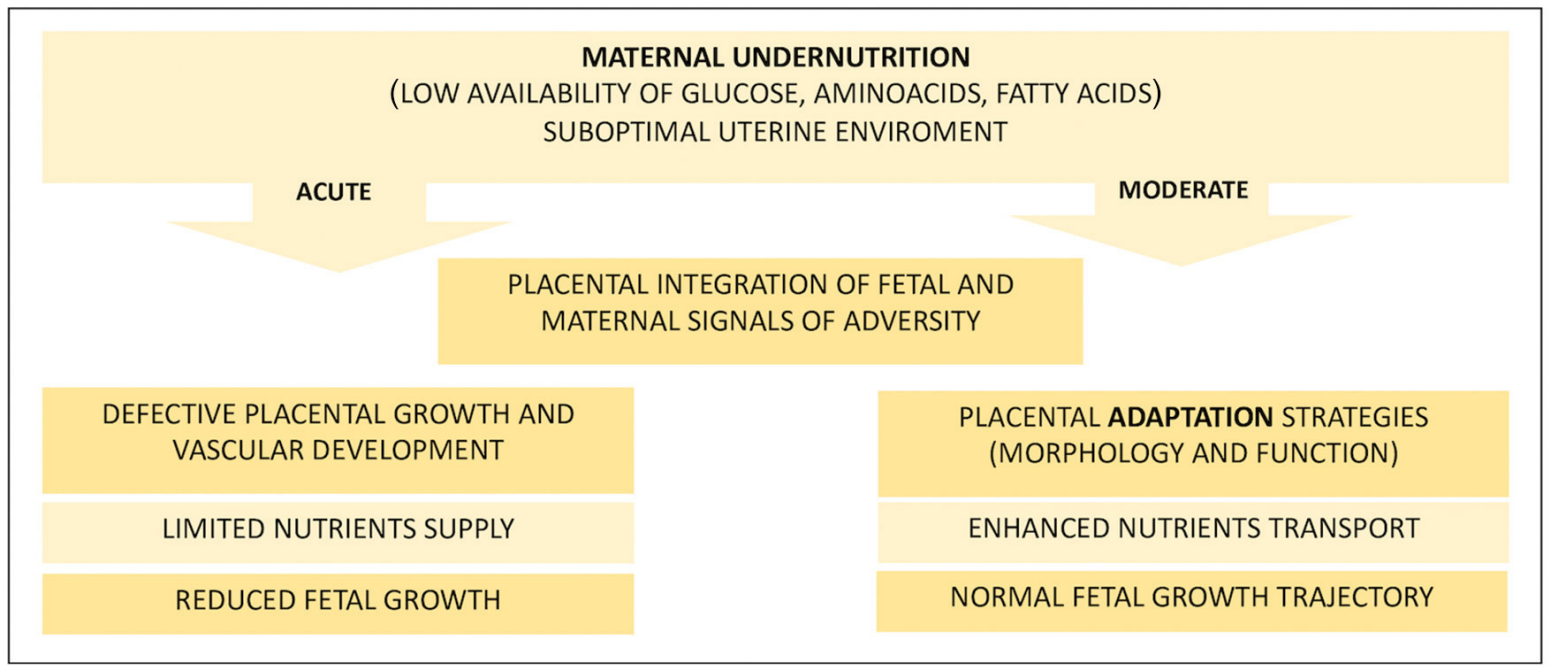
Fetal and placental effects of maternal nutritional treatments. This diagram summarizes the main events that follow an acute or moderate dietary restriction faced by the pregnant mother that affects the uterine environment. Ruminants that receive reduced maternal nutrition in early pregnancy develop a suboptimal uterine environment, and the fate of pregnancy is highly dependent on the extent of the reduction and, in turn, on the adaptability of the placenta. Acute nutritional deprivation results in defective development of the placenta, which is unable to support fetal growth with an adequate amount of nutrients. Otherwise, if the reduction of nutrients is moderate, the placenta can adapt its development by improving the transport capacity of nutrients to meet growing fetal needs and ensure the maintenance of a normal growth trajectory.

As it constitutes the center of this complicated network of autocrine and paracrine signals, the placenta cannot be considered only a passive tool for nutrient exchange between the mother and the fetus but also exerts a controlling function aimed at ensuring a balanced distribution of nutrients, which becomes particularly crucial in the case of reduced availability (see Figure 1). When nutrients are scarce, they adapt their development to maximize the use of the few available resources by enhancing the nutrient transport capacity, for example, by increasing placentome volume or vascularization density (47, 50, 51). Hence, in the case of reduced maternal nutrition, the placenta can be considered the first line of defense, especially in cases of deficiencies affecting the early phase of gestation, where the mother is programmed to accumulate nutrients and the embryo needs them as well to sustain organogenesis (52). Obviously, in the case of acute reduction of nutrients, these compensatory signals are not sufficient to satisfy the nutrient requirements of the growing fetus, which develops IUGR or, in very severe conditions, may die (18, 44).

**Figure 2:**
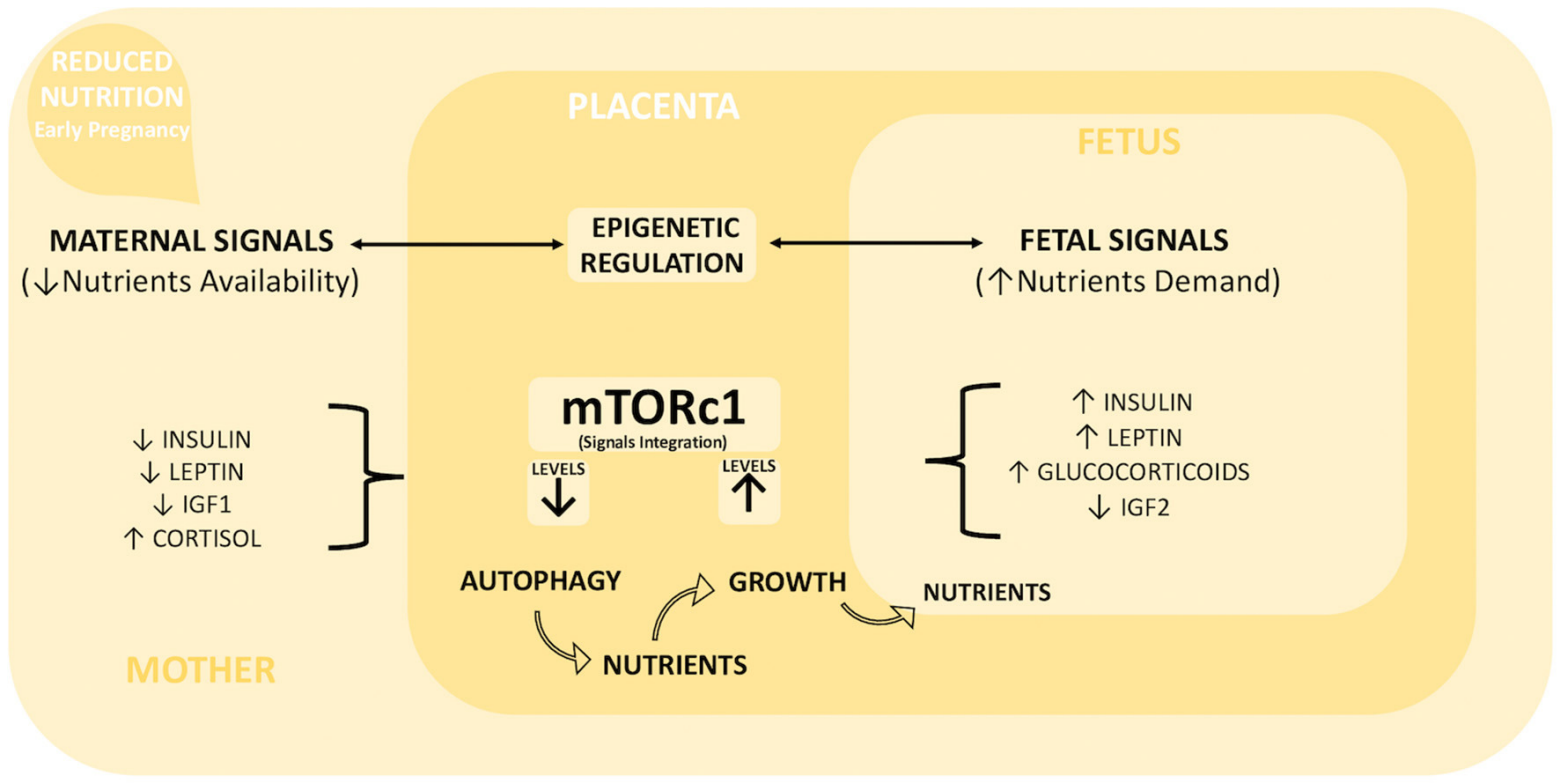
Placental integration of foeto-maternal signals. Maternal undernutrition during early pregnancy generates a series of maternal and fetal signals of adversity, whose integration is one of the key functions of the placenta. Maternal signals include low levels of insulin, leptin, and IGF1 and high levels of cortisol, while fetal signals include high levels of insulin, leptin, glucocorticoids, and low levels of IGF2. These signals are integrated by mTORc1 placental signaling. We hypothesized that moderate maternal undernutrition decreases mTORc1 levels, which in turn activate the intracellular degradation mechanism of autophagy. The degradation products can be used as new nutrients to counterbalance inadequate maternal support. This short-term increased availability of nutrients can increase mTORc1 levels and consequently enhance placental growth and nutrient transport capacity toward the developing fetus. Furthermore, the epigenetic regulation of placental gene expression (in particular on imprinted genes) plays a fundamental role for a balanced resources distribution between mother and fetus, so it could be particularly important for driving placental adaptation in the case of reduced maternal nutrition.

The placental adaptation strategies described to date following reduced maternal nutrition during early pregnancy (summarized in Table 2) seem to result from the integration of different signals derived from both the mother and fetus (32, 48, 52). The key maternal signals informing the placenta about the suboptimal nutritional status of the mother include low circulating levels of insulin, IGF1, and leptin and high cortisol levels (43, 48, 53). Following maternal undernutrition, the fetus signals to upregulate placental nutrient transport to maintain its own normal level of nutrient uptake. Usually, fetal signaling occurs via stress and other metabolic hormones (4, 37, 52), such as glucocorticoids, leptin, and insulin, although growing knowledge gives IGF2 (Insulin growth factor II) a key role in signaling fetal demands [Figure 2; (54, 55)]. Moreover, a set of placental nutrient-sensing pathways that allow integration of maternal and fetal signals has been recently identified, including adenosine monophosphate-activated protein kinase (AMPK), amino acid response-signal transduction pathway (AAR), glycogen synthase 3 (GSK-3), and mammalian target of rapamycin complex 1 (mTORc1) (32). These mechanisms of nutrient sensing enhance fetal nutrient availability by influencing maternal physiology, placental growth, and nutrient transport (56). Among them, the most relevant for our discussion is mTOR (57, 58) due to its role as an upstream negative regulator of autophagy [Figure 2; (59)].

**Table 2:** Relevant strategies of placental adaptations in ruminants following maternal undernutrition during early to mid-pregnancy.

**Model/NR length**	**Nutritional reduction (NR)**	**Placental adaptation strategy**	**References**
Sheep Days 28–78	50% of total energy intake	↑ vascular density in CAR in twins	(9)
Sheep Days 28–80 Re-feed Day 80–140	60% of energy requirements	↓ placental mass Re-feed: ↑ placental mass ↑ placental GLUT-1	(39)
Sheep Non-IUGR IUGR Day 35–125	50% of dietary requirements (NRC)	Non-IUGR: ↑ amino acid transporters IUGR: Alterations of placentome architecture ↓ total placentome volume ↓ area of the feto-maternal interface	(47)
Sheep Days 83–90 Re-feed Day 90–135	Reduced concentrate ration (n.a.)	↓ IGFBP-3 and VEGF expression ↓ placental weight ↓ number of placentomes Re-feed: ↓ IGFBP-2 expression Change of placentome type distribution	(40)
Cows Days 30–125 Re-feed Day 125–250	50% of dietary requirements (NRC)	↑ COT vascularity ↓ COT/CAR weights↑ growth signaling pathways (Akt, ERK1/2) in COT arteries Re-feed: ↓ COT and total placentome weights ↑ CAR vascularity and vessel number	(51)
Sheep Days 28–78 Re-feed Day 78–135	50% of dietary requirements (NRC)	↓ placental and placentomes weight ↑ nutrient transporter production and growth signaling (i.e., GLUT1, AMPK, ACC, ERK1/2) activity	(50)
Cows Days 30–125 Re-feed Day 125–22 Day 220–250	50% of energy requirements	↓ CAR and COT weight ↑ expression of vascular growth factor (sFlt-1) Re-feed: ↑CAR capillary surface density ↓ COT capillary area/number/surface density	(77)
Cows Days 0–99/198	Protein content Low/ High	↑ dry cotyledon weight ↑ trophoectoderm volume density	(76)
Sheep Days 0–70 Re-feed Day 70–135	85% of dietary requirements	Re-feed: ↑ COT growth Change in placentome type distribution	(78)
Sheep Days 30–80	50% of energy requirements	↑ abundance of small placentomes in fetal part ↓ weight of fetal part	(73)
Sheep Days 0–30 Re-feed Day 31–78	50% of dietary requirements (NRC)	Re-feed: ↑ vascularity in CAR and COT ↑ growth signaling pathway (Akt and ERK1/2) in COT arterial	(81)
Sheep Days 22–45 Days 45–90 Days 90–135	70% of dietary requirements	Re-feed (90–135): ↓ placental and placentome weight Change in placentome type distribution ↑ IGFBP-6 expression in the maternal villi	(89)

*Different nutritional treatments generates a series of placental developmental adaptations in order to increase the efficiency of the placental transport. Although following different routes, in most case these alterations are able to maintain fetal growth trajectory within the normal range. NRC, national research council; CAR, caruncles; COT, cotyledons; GLUT1, glucose transporter 1; IGFBP-2,−3,−6, insulin growth factor binding protein 2−3−6; VEGF, vascular endothelial growth factors; AMPK, 5′ adenosine monophosphate- activated protein kinase; ACC, acetyl-CoA carboxylase; ERK1/2, the extracellular signal-regulated kinase 1/2; sFlt-1 or sVEGFR-1, soluble fms-like tyrosine kinase-1*.

mTOR is a serine/threonine kinase that exists in 2 distinct forms (complexes 1 and 2), both of which regulate cellular adaptation to nutrient stress by coupling signals between nutrients and growth factors. mTORc1 is the major complex studied in mammalian placentas, and its activity is known to be mediated by the phosphatidylinositol 3′-kinase (PI3K)/Akt and MAPK/ERK (Mitogen-activated protein kinase/Extracellular signal-regulated kinase) signaling pathways (60).

Signal transduction from a stimulus to the regulation of cellular processes involved in the regulation of cellular homeostasis is primarily dependent on the activation of protein kinase cascade. Both the PI3K/Akt and MAPK/ERK pathways are important intracellular signal transduction cascades, regulating cell survival, differentiation, proliferation, metabolism, and motility in response to extracellular cues (61). Akt is the primary mediator of the PI3K induced cascade responsible for the activation of several downstream effectors such as mTOR. Whereas ERK, at the end of MAPK signaling cascade, is the unique substrate and downstream effector of the mitogen-activated protein/extracellular signal-regulated kinase (MEK) (62). Activation of both ERK and Akt occurs through phosphorylation and mTORC1 is a key integration points that receive many inputs from both signaling, depending on the limitation or imbalance of amino acid, glucose, insulin, and insulin-like growth factor concentrations (62–65). In particular, mTOR-dependent regulation of placental homeostasis affects both maternal and fetal IGF axes by modulating IGF binding protein 1 (IGFBP-1) expression and activity (66, 67). Interestingly, some reports proposed that placental mTORC1 is a critical hub for the homeostatic control of foeto-placental growth, adjusting the fetal growth trajectory according to the ability of the maternal supply line to provide nutrients through the placenta (60). However, no implications for the autophagy mechanism were considered in this model.

One alternative mechanism allowing cell-communication during pregnancy could involve the release and uptake of extracellular vesicles, able to transfer biological material (mRNAs and miRNAs) between cells or tissues. Despite reports on human normal and complicated pregnancy (68) recognize to the exosome-mediated delivery of miRNAs, an important roles in trophoblast physiology and foeto-maternal communication (69, 70). To date, little is known about exosomes and miRNAs during pregnancy in livestock. Exosomal miRNAs isolated from sheep uterine luminal fluid before implantation, suggest a role in conceptus-maternal communication during early pregnancy (71). Another study analyzed expression levels of different miRNAs isolated from maternal circulation, umbilical cord serum, and placentomes from early to mid-pregnancy. Interestingly, pathway analysis predicted that differentially expressed miRNAs target important pathways for cellular growth and organs development (72). However, additional works are needed to explore the role of placental miRNAs in maternal-fetal communication and their possible implication in the placental adaptation strategies to a suboptimal uterine environment.

## Effects of Reduced Maternal Nutrition During Early Pregnancy in Livestock

In the livestock supply chain, the priority of the feeding program is to provide the least expensive diet to reduce the annual budget with minimal impact on animal performance and productivity. However, when pregnancy occurs, if the nutrient supply is not adequate to sustain both maternal and fetal requirements, the mother will start to utilize its own reserves, leading to a reduction in the body condition score (BCS), which is a crucial determinant for optimal pregnancy proceedings (73).

Worldwide, nutrient deficiency has been reported to occur very often in animals provided with forage-based diets due to its seasonal variation in both quality and quantity. For example, in Australia (southwestern Queensland), the quality of cattle nutrition is strongly influenced by seasonality, with a very low (5% instead of 15%) crude protein (CP) concentration during the winter period (11, 74). Similarly, in the western region of the United States (Montana), the nutrient uptake of grazing ewes is often ~50% of their NRC (National Research Council) nutrient requirement due to the poor quality of forage (17). In such situations, if pregnancy occurs without any kind of diet supplementation (16, 75), fetal growth will be seriously compromised, probably due to defective placentation. In fact, several nutritional experiments conducted on ruminants have demonstrated alterations in different aspects of placental development and transport in response to reduced maternal nutrient availability [Table 2; (8, 40, 50, 51, 73, 74, 76–81)]. However, a critical analysis of the results is complex due to the high variability of the experimental conditions applied in both sheep and cows, including diet composition, length of treatment, and timing of evaluation. Additionally, for most nutritional studies conducted on livestock, the main purpose is to evaluate the effects of an unbalanced maternal diet on offspring health and productivity (10–12). Hence, placentas are often analyzed at the end of pregnancy, giving us an incomplete picture of what occurred during early development, especially in cases where fetal growth is offset and placental defects are no longer visible (50, 51).

Nevertheless, the development of the placenta is more susceptible to nutritional depletions that affect early and mid-pregnancy, the phase of rapid placenta growth (8, 13, 24). In general, most studies in livestock show that severe undernutrition reduces placental growth, whereas less extreme reductions in the maternal diet have the opposite effect (2) (Figure 1). For example, in sheep, moderate (−15%) nutrient restriction from early to mid-pregnancy (0–70 d) alters placental morphology at term, showing increased growth of the fetal side of the placentome (78). However, because fetal growth follows the normal trajectory, this can be considered a sign of adaptation rather than a developmental defect. Whereas, ewes sustain more severe conditions (50–60% of ER), even for a short period (30–80 days), with a decrease in the placental weight at mid-gestation, a higher number of placentomes have been observed in comparison with adequately fed controls (73). Interestingly, if the effect of a similar treatment was analyzed at full-term pregnancy (79), an increased total placental weight was observed, indicating that a higher number of placentomes successfully enhanced nutrient uptake later in pregnancy when normal nutrition was restored. Again, a more significant response to the fetal part has been reported, suggesting a greater response of this placental portion to nutritional deficiencies (73, 78). At mid-gestation, reducing (−30%) only the CP of the diet causes a decreased proportion of large placentomes in favor of a high number of small placentomes with a predominant vasculature, compared to sheep offered the same reduction of the total diet (79). This suggests that protein availability preferentially affects the vascular compartment of the placenta, as supported by the observation that a low CP diet in the first trimester of bovine pregnancy increased the blood vessel volume and volume density of fetal villi at term (8).

Similarly, many studies in sheep and bovines have shown that the placenta alters its vascular development to minimize the impact of reduced nutrient availability on fetal growth (Table 2). The reported changes following different protocols of nutrient restriction include modulation of cotyledon vascularity (51), early placentomal differentiation (80), or upregulation of the expression of angiogenetic factors (77). For example, in sheep, it has been reported that the placenta reacts to maternal undernutrition (50%) imposed until day 30 of gestation by increasing the vascular density of the placentomes, despite their reduced average weight at mid-gestation (81). This adaptation strategy maintains the trajectory of fetal growth within a normal range and occurs in association with upregulation of the MAPK/ERK1/2 and PI3K/Akt growth signaling pathways in cotyledonary arterial tissue of placentomes, which is expected to promote placental growth and angiogenesis (81).

Similarly, cows that were nutrient restricted (50%) from early to mid-pregnancy (30–125 d) exhibited increased cotyledon vascularity, improved placentome efficiency and increased phosphorylated Akt and ERK1/2 in cotyledonary arteries compared to controls (51); however, all treatment-related differences detected in undernourished cows were lost at term.

In sheep, Ma et al. (50) demonstrated that maternal nutrient reduction (50%) from early (28 d) to mid-gestation (78 d) stimulates the placenta to activate several mechanisms that increase placental transport capacity and, in turn, fetal growth. These mechanisms include upregulation of glucose and fatty acid transporter expression, increased activity of AMPK and ERK1/2, and upregulation of angiogenesis in cotyledonary tissues from mid-gestation onward. At that time, both fetal weights and length were significantly lower in nutrient-restricted ewes, indicating that restriction of the maternal diet induced fetal growth reduction. After refeeding, due to placental compensation, restricted fetuses exhibited a catch-up growth rate and reached similar weights and body lengths compared to the control fetuses in late gestation (50).

The AMPK pathway is activated by a variety of physiological stimuli, such as glucose deprivation or hypoxia, resulting in a reduction of cellular energy level and an increase in AMP/ATP ratio (61). It is interesting to note that, together with the others metabolic related signaling pathways mentioned above (Akt, ERK1/2), is also well-documented mediator of autophagy activation (19); however, no direct observations on autophagic flux have been clearly documented in relation to placental adaptation strategies.

Interestingly, more efficient placental responses to nutritional depletion (50%) have been reported in ewes habitually managed under harsh environments with limited food availability. These placentae displayed an early conversion of placentomes with an increase in size and vascularity. In contrast, this conversion failed to occur in control ewes, suggesting that placentae adapted to growth in a suboptimal environment are more prone to overcome a stressful situation by using more efficient strategies than those facing this situation for the first time (80).

At the cellular level, previous stressor experienced by mother cell as well as previously adopted contrasting strategies can be communicated to the descendant cells, to better adaptation. Inheritance of previous stress responses in a “memory” -like manner can be expected to serve as a mechanism to enhance cell survival and can occur through epigenetic regulatory mechanisms, allowing cells to rapidly switch gene expression patterns or growth states in response to adverse environmental conditions (82). However, epigenetic studies to deeply define the mechanism are still poorly developed in mammals.

## The Potential Involvement of Epigenetic Regulation

The concept of fetal programming has been introduced to explain the role of developmental plasticity in response to environmental and nutritional signals during intrauterine life and its potential negative consequences (risk of cardiovascular, metabolic, and behavioral diseases) in postnatal life (4). Although the initiation mechanism remains unclear, recent observations in humans and rodents suggest that epigenetic changes in regulatory regions and growth-related genes play a significant role in this adaptive process involving both the fetus and the placenta (6).

The disorders associated with suboptimal foeto-placental growth are frequently caused by changes in the regulation of the IGF axis, which operates via two mitogenic polypeptides, IGF1, and IGF2, whose specific action and bioavailability are modulated by IGF binding proteins (IGFBPs) and IGF receptors (IGFRs) (67, 83). As discussed above, knockout mice revealed a role for IGF2 as a key fetal signal able to modulate placental growth and amino acid transport (84). In mice lacking the placental-specific IGF2 transcript, placental growth is compromised starting from early gestation, but normal fetal growth is maintained until late gestation (85). This occurs due to increased placental transport of glucose and amino acids associated with upregulated expression of genes belonging to the System A amino acid transporter gene family (85). Interestingly, if these mice were subjected to maternal undernutrition, IGF2-deficient placentas failed the compensatory upregulation of those genes in response to nutrient restriction (86). In ruminants, the IGF axis is thought to play a fundamental role in placental and fetal growth as well as the ability to regulate metabolism, nutrient partitioning, and nutrient transport (87, 88). Several studies report that the synthesis of the components of the IGF system can be modulated with different outcomes through nutrition. For example, in the sheep placentome, IGF2 mRNA expression seems to be unaffected by maternal undernutrition (45–90 d), notwithstanding the increase in placental weight (89). However, IGF2 activity could be indirectly modulated, as this and other authors reported alterations in the expression of IGFBPs, which are thought to inhibit actions of IGF2 by reducing their bioavailability, following maternal undernutrition (40, 74, 89). Similarly, as IGF2R would be expected to increase IGF2 turnover and reduce its anabolic actions (83), the reduced IGF2R expression detected in rat placenta following a low CP diet could partially explain the impaired placental growth as well as the retarded fetal growth found in this model (90). The role of IGF2 in regulating foeto-placental growth is also supported by pioneering studies on murine models exploring the epigenetic mechanism involved in the regulation of gene expression during mammalian development (91, 92). Among these, genomic imprinting has been identified as the mechanism regulating the monoallelic expression of genes in a parent-of-origin-specific manner, resulting in their functional differences during development (93). Silencing of the unexpressed allele occurs by epigenetic marks, including DNA methylation, which are known to be influenced by environmental factors and nutrients (94, 95). Such a regulated gene cluster, called imprinted genes, displays strong placental expression, and is thought to be involved in both fetal and placental growth mainly via the regulation of nutritional resource allocation (84). In particular, the parental conflicting theory (96) proposes that paternally expressed genes support the extraction of maternal resources for the benefit of the fetus; in contrast, maternally derived genes tend to counteract this effect to prevent complete depletion of resources for the pregnant mother (Figure 2). IGF2 is one of the most studied imprinted genes, which, paired with another well-known imprinted gene, lncH19 (long non-coding H19), regulates placental growth and nutrient transport capacity, playing opposite roles (97–99). Even the type 2 receptor gene (IGF2R) necessary to prevent overabundance of IGF2 is reported to be imprinted (100, 101), as well as genes for placental amino acid transporters [Slc38a4 (78)] or genes regulating maternal nutrient partitioning (Paternally Expressed Gene PEG3) (102), suggesting that imprinting mechanisms can be implicated in the developmental reprogramming of placental transport under a suboptimal nutritional environment. Unfortunately, few reports are currently available on the imprinted status of placental genes from undernourished mothers, and most have conflicting data (103–105). For example, one study reported that the methylation pattern of placental lncH19 and IGF2 was unaltered in response to maternal protein restriction in rats, even if IGF2 expression was often affected (103).

In contrast, another report conducted in mice showed that maternal caloric restriction that results in IUGR differentially affects placental gene expression and whole genome DNA methylation in a sex-specific manner (male). In particular, a set of differentially methylated imprinted genes and miRNAs targeting genes responsible for transplacental nutrient transfer was identified, suggesting specific susceptibility of these epigenetic marks to nutritional depletion (104).

Furthermore, by examining the broad gene expression profiles of fetal and placental tissue of mice undernourished *in utero*, some authors concluded that the expression of imprinted genes does not seem to be more susceptible to perturbation induced by maternal undernutrition than other genes (105). However, they propose that the selective modulation of certain imprinted genes (lncH19, IGF2R, and PEG3) in fetal liver plays an important role in the adaptive fetal response to *in utero* undernutrition.

On the other hand, it is conceivable that changes in maternal nutrition status alter the availability of methyl donors, influencing the global and specific methylation status of the developing placenta. Interestingly, some studies have reported nutritional influences on DNA methylation levels in specific regions of the brain (106), liver (107), or gametes (108). However, more detailed experiments are needed to establish a clear link between placental epigenetic regulation and maternal undernutrition.

## Role of Placental mTOR in the Control of Nutrient Availability

As previously discussed, mTOR is a serine/threonine kinase that controls cell growth, proliferation, and metabolism in response to nutrient variation. It forms two distinct protein complexes, mTOR complex (mTORC) 1 and 2, which have specific substrate preferences and trigger different downstream signals to regulate cell function and viability. mTORC1 responds to growth factors, energy, oxygen, and amino acids and is primarily involved in the regulation of cell growth and metabolism (60, 64, 66). It promotes anabolism by increasing the synthesis of proteins, lipids, glucose, and nucleotides and by inhibiting the catabolic process of autophagy. In contrast, mTORC2 is regulated by growth factors and controls mainly cell growth and proliferation, survival, ion transport, cell migration, and cytoskeletal remodeling through its downstream substrates, including PKC and Akt (60, 64). During pregnancy, placental mTOR plays an important role in the regulation of fetal growth in response to nutritional changes (57). Data collected from growth-restricted pregnancies (58, 66) suggest that during placental development, mTOR serves as a link between maternal nutrient availability and fetal growth. This was confirmed even in rats fed low-protein diets that showed placental mTOR inhibition in association with a downregulation of placental amino acid transporter expression and restricted fetal growth (44, 57).

Other study conducted on human trophoblast cells report that mTOR seems to act as a molecular mechanism of nutrient sensing, which integrates multiple signals indicating the presence of major substrates (such as free fatty acids, amino acids, and glucose) in the maternal circulation and responds with up- or downregulated placental growth and nutrient transporters [Figure 2; (109)]. In particular, the mTOR pathway stimulates system A and L amino acid transporters, which are critical for the transport of both essential and non-essential amino acids to the fetus (110). Moreover, mTOR indirectly regulates placental protein synthesis and active transport mechanisms by modulating ATP availability (through regulation of trophoblast oxidative phosphorylation) with expected consequences for placental growth (111).

In addition, mTOR responds to hormones and growth factors in maternal circulation and within the placenta. More specifically, it is activated by maternal insulin, IGF1 and leptin, and it is inhibited by adiponectin and cortisol (58). Inhibition of placental mTOR has been frequently reported in association with a reduction in insulin/IGF1 signaling and amino acid transporters in rats fed low-protein diets (44, 57), in mice suffering maternal folate deficiencies (112), and in human placenta from IUGR pregnancy (113). In contrast, a study conducted on ewes by Ma et al. (50) reported that maternal nutrient restriction until mid-gestation does not alter placental expression of Akt and mTOR, despite a 26% decrease in fetal weight that will be normalized later in gestation. In addition, unlike other studies demonstrating downregulated expression of amino acid and glucose transporters in IUGR placentas from human and rat (44, 58), the same study reported an increased level of glucose and fatty acid transporters at mid gestation (50). This difference in the sheep model suggests that these placentas may activate a compensatory reaction that does not involve Akt/mTOR but passes through other important regulatory pathways in the placenta, such as AMPK or ERK1/2 signaling.

Finally, mTORc1 acts as the most important negative regulator of autophagy, since inactivation of mTOR is an upstream event governing the activation of autophagic mechanisms. These degradation mechanisms serve to provide cells with free amino acids, carbon, azote, and fatty acids for survival in adverse conditions such as starvation or hypoxia. Amino acid or growth factor deprivation is the most effective stimulus inducing autophagy, and both are linked to the mammalian target of rapamycin complex 1 (mTORC1) pathway [Figure 2; (59)]. As demonstrated using different cell culture systems (MEF, HEK293, and HEK293T), under normal conditions mTORC1 phosphorylates components of the Unc-51-like kinase 1 (ULK1) complex to prevent autophagy initiation. Interestingly, recent studies have shown that mTOR and AMPK coordinate the regulation of cellular nutrient and energy signals to maintain cellular homeostasis through phosphorylation of ULK1 at distinct serine residues. Under conditions of cellular energy deficiency, AMPK phosphorylates ULK1 to disrupt the interaction with mTOR, thus resulting in autophagy activation (114).

## Signs of Autophagy in Placental and Trophoblast Cells Subjected to Adverse Conditions

Autophagy is an intracellular, self-degradation system necessary to maintain cellular homeostasis during critical periods of cellular differentiation and tissue development and in response to nutrient stress. It is characterized by the engulfment of cytoplasmic material, aggregated proteins, and organelles into a double-membraned structure (autophagosome) that is able to degrade the sequestered materials and recycle the obtained products to increase nutrient availability for the suffering cell (19). What makes this mechanism so interesting is the ability to self-increase its activity to protect cells from nutritional deficiencies or in response to altered metabolism. The surveillance of cellular homeostasis under low nutrient availability is a crucial step for the maintenance of cell survival, and autophagy is a mechanism that supports this purpose (19).

Several signaling pathways underlying the regulation of cell autophagy have been identified, including the PI3K/Akt/mTOR, MAPK/ERK/mTOR, and AMPK signaling pathways that promote cell growth, proliferation, and survival (114). Moreover, autophagy regulation by amino acid and glucose availability has been extensively validated in different cellular systems. In particular, when cells suffer amino acid starvation, mTOR is inactivated, leading to activation of autophagic signaling to increase amino acid availability by degrading proteins. Similarly, in the case of glucose starvation, AMPK will be activated immediately to prevent ATP consumption and increase glucose intake to maintain energy homeostasis (114). At the same time, the autophagic pathway is activated to recycle nutrients (19). However, this process is not unlimited, and beyond a certain threshold, it can become harmful to the cell and even cause death.

Recent data obtained from human and murine studies indicate that during mammalian pregnancy, autophagy plays an essential role in the development of the placenta (115) as well as in the regulation of resource allocation (116), which has spurred further studies on the involvement of autophagy in placental dysfunction (117–119).

Given that the characterization of this cellular mechanism is relatively recent, most of the knowledge about it derives from genetic manipulation studies in mice or from observations in the human clinic, whereas *in vitro* cell systems are mainly used for testing both upstream and downstream signaling pathways involved in the regulation of autophagic flux during placentation (Table 1). In trophoblast cells obtained from human, mouse, and pig placentas, autophagy activation has been tested *in vitro* using different stress culture conditions or chemical compounds, confirming a role for autophagy in controlling essential trophoblast mechanisms such as cell differentiation, proliferation, migration, and invasion (120–125).

In mice subjected to different deficient diets, autophagy has been reported to play multiple important roles in embryonic development, trophoblast functionality, and placenta formation, although the governing mechanisms are still partially unknown (126, 127). Furthermore, abnormal increases in autophagic activity have been reported even in gestational diseases sharing a common origin on defective placental development, such as preeclampsia (21, 117) or intrauterine growth restriction (118, 119). Interestingly, evidence of placental autophagy has been found in pregnancies complicated folate deficiency (126), in agreement with data obtained from folate-deficient mice, which develop IUGR in association with inhibition of placental mTORC signaling and decreased amino acid transporter expression and activity (112).

As folate influences the availability of methyl donors, these reports suggest a possible link between placental autophagy and maternal undernutrition, which can result in the unbalanced regulation of the methylation machinery and in turn affects the expression of regulated genes, including imprinted genes. However, this fascinating line of research is still poorly explored, with very few reports looking at the placental epigenetic profile following a period of reduced maternal nutrition.

An indirect link connecting autophagy regulation with genomic imprinting has been provided by data obtained from autophagy inhibition in trophoblast cells. Specifically, experiments conducted on humans and mice demonstrated that autophagy suppression seems to induce spontaneous abortion by stimulating IGF2 secretion and PEG10 (Paternally Expressed Gene 10) reduction. Furthermore, high IGF2 levels lead to decidual NK cell differentiation and cytotoxicity activation at the maternal-fetal interface, while decreased PEG10 reduces the invasiveness of trophoblast cells, contributing to early pregnancy failure (121). Moreover, silencing the imprinted gene lncH19, which is known to be downregulated in human placentae from fetal growth restriction (FGR) pregnancies, inhibited the proliferation and invasion of trophoblast cells and promoted autophagy by targeting miR-18a-5p (128). Accordingly, overexpression of lncH19 promotes invasion and autophagy activation via the PI3K/Akt/mTOR pathway in human trophoblast cells, confirming the crucial involvement of this gene in trophoblast cell functionality (129).

Few studies thus far have reported the role of mTOR in ruminant pregnancy. In goats, the CREBRF (CREB3 Regulatory Factor)-mTOR-autophagy pathway plays a central role in prostaglandin secretion and cell attachment in regulating endometrial function (130). In ewes, inhibition of angiogenesis also resulted in reduced activation of Akt/mTOR signaling and elevated LC3B-II, a marker of cellular autophagy in the endometrium. This study suggests that the CXCR4 (C-X-C Motif Chemokine Receptor 4) signaling at the ovine fetal–maternal interface governs placental homeostasis by serving as a critical upstream mediator of vascularization and cell viability, thereby ensuring appropriate placental development (131).

Increased placental autophagy has been reported in early (20 days) ovine placenta obtained from embryos produced *in vitro* (132). Interestingly, these placentae are characterized by reduced vessel development (30) as well as by reduced expression of the imprinted genes lncH19 and IGF2 (133); however, no data on mTOR or other regulatory pathways have been provided.

Other evidence that indicates an important role for placental autophagy in supporting fetal growth came from interesting work demonstrating that in mice, even short-term food deprivation produces significant changes in hypothalamic and placental gene expression (20). In particular, 24-h starvation decreased PEG3 imprinted gene expression in the placenta in association with increased autophagy and ribosomal turnover. Interestingly, such dysregulation does not occur in the hypothalamus, where PEG3 expression increases following food deprivation. Hence, normal brain development seems to be maintained at a cost to its placenta, which sustains, at least for the short term, nutrient supply for the developing hypothalamus (20).

Similarly, using a murine model, it has been shown that short-term (48 h) food deprivation causes mTOR deactivation in starved placentae in association with an increase in the autophagic marker LC3B compared to basal levels in controls (127).

A possible connection between placental vascularization and autophagy has been explored through the study of soluble decorin, a pan-receptor tyrosine kinase inhibitor that affects the biology of several receptor tyrosine kinases by triggering receptor internalization and degradation. It is involved in the regulation of autophagy and inhibition of angiogenesis in both microvascular and macrovascular endothelial cells. This process is mediated by a high-affinity interaction with VEGFR2 (vascular endothelial growth factor receptor), which leads to increased levels of the maternally imprinted gene PEG3 (134, 135). Autophagy during the process of placental vascularization has been demonstrated to occur even during initial placentation in sheep (20 days), when dysregulation of central placental chemokine signaling (CXCL12-CXCR4 axis) at the fetal–maternal interface leads to decreased local vascularization and suppressed Akt/mTOR signaling and promotes induction of autophagy, with further implications for proper placentation (131). Moreover, a recent study using a rat model of immune-mediated pregnancy loss proposed drug-induced placental autophagy as a good candidate for therapy due to the resulting improvement in pregnancy outcomes. Following placental autophagy induction, associated with downregulated mTOR placental expression, the study reported an increased fetal weight and decreased resorption rate due to suppression of placental inflammation (136).

Finally, in a murine model of IUGR (137) obtained with mild to moderate late gestational food restriction, it was reported that autophagy and Endoplasmic Reticulum stress pathways are decreased in placentas from growth-restricted mothers, especially in the junctional zone of the murine placenta. This is in contrast to most of the data from both *in vivo* and *in vitro* studies (20, 112, 126, 127) that report the activation of autophagy following nutrient deficiency; however, due to the peculiar ability of the placenta to adapt its own development according to variations in the intrauterine environment, it is possible to argue that this organ may also have the ability to guide the direction of the autophagic response by taking into account several factors, such as the general well-being of the mother or the timing and severity of nutritional deprivation.

## Conclusion

The role of placentation in achieving efficient reproductive success has not yet been sufficiently investigated in livestock despite the evident impact that environmental and nutritional conditions may have on fetal growth and the development of possible alterations subsequent to the fetal phase that affect performance growth in offspring.

This review has highlighted the studies carried out to examine nutritional deficiencies in ruminants and shows the results obtained with laboratory species and humans, where this problem has historically been addressed for the longest time.

In particular, we have deepened a very intriguing aspect of the role of autophagy as a homeostatic mechanism that seeks to compensate, within certain limits, for external influences on intrauterine growth. Its regulation through general hormonal pathways could also be central in supporting and improving the placenta-fetus interaction and in achieving reproductive success, in livestock. The role of mTOR in ruminants is proposed to be central to understanding this regulation, although in these species, this intracellular pathway has been elucidated in few studies to date. Via mTOR, autophagic regulation interferes with placentation, such as the vascularization process and the regulation of imprinted genes. The definition of which main pathway is used will determine effective and improved strategies for the management of pregnancies, even in livestock supply chains with the most exposure to nutritional and energy alterations during pregnancy.

## Author Contributions

PT and MB wrote sections of the manuscript. All authors contributed to manuscript revision, read, and approved the submitted version.

## Funding

This work was supported by local grant UNITO 2020, cofund Department of Excellence (TOSP_RILO_20_01) and Smartsheep project, 2018-21Agricoltura 4.0, Fondazione CRC.

## Conflict of Interest

The authors declare that the research was conducted in the absence of any commercial or financial relationships that could be construed as a potential conflict of interest.

## Publisher's Note

All claims expressed in this article are solely those of the authors and do not necessarily represent those of their affiliated organizations, or those of the publisher, the editors and the reviewers. Any product that may be evaluated in this article, or claim that may be made by its manufacturer, is not guaranteed or endorsed by the publisher.
